# How Do We Decolonize Global Health in Medical Education?

**DOI:** 10.5334/aogh.3220

**Published:** 2021-03-24

**Authors:** Deen L. Garba, Makela C. Stankey, Anusha Jayaram, Bethany L. Hedt-Gauthier

**Affiliations:** 1 Program in Global Surgery and Social Change, Harvard Medical School, Boston MA, US; 2 University of North Carolina School of Medicine, Chapel Hill NC, US; 3 Keck School of Medicine at the University of Southern California, Los Angeles CA, US; 4 Tufts University School of Medicine, Boston MA, US; 5 Harvard Medical School, Department of Global Health and Social Medicine, Boston MA, US

## Abstract

Medical schools are increasing global health training opportunities, but these have been marketed to medical students as an exotic vocation. The challenges of global health education in high income country (HIC) medical schools are rooted within broader inequities in global health partnerships. More meaningful engagement during medical training is hindered by students’ inability to take extended absences, difficulty securing funding, a paucity of mentors with demonstrated commitment to equitable global health practice, and inadequate preparation. Calls for decolonizing global health have recently amplified, and medical schools must seize the opportunity to train decolonizers. We outline steps medical schools can adopt to shift their global health education approach to develop practitioners better prepared to contribute equitably. First, students should be exposed to more global health courses, including the history of colonial medicine and its effects on specific local contexts. Medical schools should deemphasize short-term unidirectional engagement, and encourage extended experiences. International experiences must have clearly defined roles, clarified with pre-visit contracts and supervision of the experience to ensure students do not engage in medicine above their level of training. For any exchange, medical schools must provide pre-visit training that includes site-specific orientation and strategies for effective collaboration. Finally, medical schools must recruit faculty committed to developing equitable, long-term collaborations, and institutional promotion criteria must be aligned to encourage this work. An understanding and commitment to this lifelong practice can be fostered through medical school curricula that expose students to global health work that prioritizes equity in clinical work and research.

In response to more interest among students, medical schools are increasing the global health training opportunities available [1]. However, many of the current strategies to expose medical students to global health are fraught, including an emphasis on transient cultural immersions, suboptimal pre-visit training, and poorly defined student roles for engagement [2,3]. Combined, these undermine the student’s learning and any potential benefit to the host institution. The challenges of global health education in high-income country (HIC) medical schools are rooted within long-standing, broader inequities in global health partnerships. Current models provide little understanding of the legacy of colonization in global health initiatives, or of how power imbalances due to colonialism, racism, and epistemic injustice continue to shape inequitable relationships in global health practice and research.

While some students are motivated to pursue global health opportunities because they anticipate a future career in the global arena, it is not uncommon for medical schools to market these opportunities as an honorable vacation, résumé item, or residency application talking point [4]. Furthermore, some trainees perceive global opportunities, such as pre-med experiences marketed by for-profit companies, as an opportunity to engage in a greater level of clinical work than one would in their home institution, thereby placing patients’ and students’ health at risk [5]. As highlighted in Lasker et al., these short-term engagements create a false sense of normalcy with dangerous implications [6]. However, more meaningful engagement during medical training is hindered by students’ inability to take extended absences, difficulty securing funding, inadequate preparation for these experiences, and a paucity of mentors with demonstrated commitment to equitable global health partnerships [7].

Colonization in global health refers to the enduring legacy of colonial structures and power differentials that drive discrimination and allow for disproportionate benefits to individuals at HIC institutions at the expense of their low- and middle-income country (LMIC) partners [7]. Costs to the host institutions can include a distraction from the institution’s priorities, increased use of physical resources, stretched responsibility to provide oversight to new students, and a lack of reciprocal opportunities [8,9]. Calls for decolonizing the field of global health have been amplified over the last few years, and US medical schools must seize the opportunity to train decolonizers – students who understand the complicated framework and history of global inequities and seek action-oriented paths that work in the best interest of the communities they wish to impact.

As a group of senior medical school students who have committed to integrating global health into our long-term career plans, we have supplemented our own education with the goal of decolonizing our approach to global health engagement. We believe students in medical education should pursue their interests in health equity in global spaces to their fullest potential, and at the same time, we recognize our risk of perpetuating colonial ideas despite our good intentions. From our experiences, we outline steps that can be adopted by US medical schools to shift their global health education approach to develop practitioners better prepared to contribute equitably.

First, medical students should be exposed to more global health courses, including the history of colonial medicine and its effects on specific local contexts. These courses should be supplemented with training in global health ethics, health equity, cultural sensitivity, and building partnerships, such as the recommended strategies for developing partnerships that are sustainable, ethical, and beneficial to host communities outlined in the Brocher Declaration [10]. Courses rooted in these concepts may mitigate romanticized notions medical students have regarding global health, such as “fixing” a country’s problems. These courses also create space for students to challenge their standards in health equity, critically reflect on their motivation to enter global health, recognize their benefits from participating, and consider their impact – both positive and negative – in any global health exchanges. These competencies will be foundational for those who go on to pursue careers at the intersection of global health and medicine.

Medical schools should also deemphasize short-term, unidirectional engagement. Extended experiences should be encouraged, such as gap years in which students participate in global health fellowships. Such prolonged commitments demonstrate a willingness to center the cause above one’s individual professional advancement. Through established long-term partnerships at the institutional level, short-term exchanges at international sites can remain an important part of global health opportunities in medical schools. Continuity must be coordinated for effective student-to-student handoff. All international experiences should be linked to the host institution’s priorities, must have defined roles clarified with pre-visit contracts co-developed with the host institution, and have close supervision.

For any type of exchange, medical schools must provide pre-visit training that includes site-specific orientation and strategies for effective collaboration to ensure that students are sufficiently prepared. Importantly, medical schools must have strong oversight to ensure students who travel to international sites do not practice medicine above their level of training. Students interested in global health should be encouraged to connect with faculty and identify a project early in their training, and should stay engaged in those projects for the full tenure of their medical school training, even when not physically present at the international sites.

Medical schools must recruit faculty committed to developing equitable, long-term collaborations with global health partners, and the institution’s faculty promotion criteria must be aligned so as to encourage this work [11]. These faculty are the custodians of partnerships where power dynamics can be broken down to allow for bi-directional student visitation, dedicated research funding streams, and ongoing needs assessments to ensure visiting students do not strain local resources. Importantly, these faculty members are also essential in helping to build the medical student’s research capacity grounded in contextual understanding. Medical schools can also promote decolonization in global health research by requiring faculty to involve partner organizations in all aspects of international research, achieve equity in collaboration such as sharing key resources and fair authorship practices, and link research to advocacy. Lastly, medical schools should establish institutional task forces that hold students and faculty accountable in centering equity in all global health endeavors.

We aspire to promote a generation of socially conscious physicians better prepared to care for an increasingly diverse global citizenry, no matter where they practice in the world. Decolonizing global health will not occur overnight. US medical schools can foster an understanding and commitment to this lifelong practice, exposing students to global health work as a long-term pursuit that centers on equity in clinical work and research.

## References

[1] Göpfert A, Mohamedbhai H, Mise J, et al. Do medical students want to learn about global health? Glob Health Action. 2014; 7: 23943. DOI: 10.3402/gha.v7.23943 24848658 PMC4028928

[2] Bruno DM, Imperato PJ. A global health elective for US medical students: The 35 year experience of the State University of New York, Downstate Medical Center, School of Public Health. J Community Health. 2015; 40(2): 187–198. DOI: 10.1007/s10900-014-9981-0 25564184 PMC7087751

[3] Bauer I. More harm than good? The questionable ethics of medical volunteering and international student placements. Trop Dis Travel Med Vaccines. 2017; 3(5). DOI: 10.1186/s40794-017-0048-y PMC5531079 28883975

[4] Stoltenberg M, Rumas N, Parsi K. Global health and service learning: Lessons learned at US medical schools. Med Educ Online. 2012; 17. DOI: 10.3402/meo.v17i0.18848 PMC3401288 22826631

[5] Jacquet GA, Sarfaty S, Tupesis JP. Comments on “Protecting the health of medical students on international electives in low-resource settings.” J Travel Med. 2018; 25(1). DOI: 10.1093/jtm/tay008 29608737

[6] Lasker JN, Aldrink M, Balasubramaniam R, et al. Guidelines for responsible short-term global health activities: Developing common principles. Global Health. 2018; 14(1): 18. DOI: 10.1186/s12992-018-0330-4 29415740 PMC5803894

[7] Eichbaum QG, Adams LV, Evert J, Ho M-J, Semali IA, van Schalkwyk SC. Decolonizing global health education: Rethinking institutional partnerships and approaches. Acad Med. Published online 4 28, 2020. DOI: 10.1097/ACM.0000000000003473 32349015

[8] Bozinoff N, Dorman KP, Kerr D, et al. Toward reciprocity: Host supervisor perspectives on international medical electives. Med Educ. 2014; 48(4): 397–404. DOI: 10.1111/medu.12386 24606623

[9] Renaud-Roy E, Bernier N, Fournier P. Host perspective on academic supervision, health care provision and institutional partnership during short-term electives in global health. Med Educ. 2020; 54(4): 303–311. DOI: 10.1111/medu.14027 31875656

[10] Brocher Declaration – ICMDA. Accessed 2 10, 2021. https://icmda.net/about/partners/brocher/.

[11] Hedt-Gauthier B, Airhihenbuwa CO, Bawah AA, et al. Academic promotion policies and equity in global health collaborations. Lancet. 2018; 392(10158): 1607–1609. DOI: 10.1016/S0140-6736(18)32345-6 30496066

